# Advances and Opportunities of Mobile Health in the Postpandemic Era: Smartphonization of Wearable Devices and Wearable Deviceization of Smartphones

**DOI:** 10.2196/48803

**Published:** 2024-01-22

**Authors:** Wonki Hong

**Affiliations:** 1 Department of Digital Healthcare, Daejeon University, Daejeon, Republic of Korea

**Keywords:** mobile health, mHealth, smartphonization, wearable deviceization, new form factor, sensor-integrated display

## Abstract

Mobile health (mHealth) with continuous real-time monitoring is leading the era of digital medical convergence. Wearable devices and smartphones optimized as personalized health management platforms enable disease prediction, prevention, diagnosis, and even treatment. Ubiquitous and accessible medical services offered through mHealth strengthen universal health coverage to facilitate service use without discrimination. This viewpoint investigates the latest trends in mHealth technology, which are comprehensive in terms of form factors and detection targets according to body attachment location and type. Insights and breakthroughs from the perspective of mHealth sensing through a new form factor and sensor-integrated display overcome the problems of existing mHealth by proposing a solution of smartphonization of wearable devices and the wearable deviceization of smartphones. This approach maximizes the infinite potential of stagnant mHealth technology and will present a new milestone leading to the popularization of mHealth. In the postpandemic era, innovative mHealth solutions through the smartphonization of wearable devices and the wearable deviceization of smartphones could become the standard for a new paradigm in the field of digital medicine.

## Background

In the postpandemic era, the significance of mobile health (mHealth) has been highlighted, and explosive growth in this area is expected to continue [1,2]. Cutting-edge technologies are converging with health care, and mHealth, based on hyperconnected intelligence, is leading the paradigm shift in medical care [3,4]. Many countries have already entered a superaged society, and the proportion of gross domestic product expenditures for medical care is increasing due to an upsurge in the number of people with chronic diseases. In addition, the excessive demand compared to the available supply, the lack of health care infrastructure, and the unbalanced distribution of medical staff are also problems. Therefore, prediction, prevention, and management through artificial intelligence (AI)–based medical big data analysis are required, and for this purpose, ubiquitous and accessible medical services using personalized devices must be provided [5]. mHealth is a strong candidate to make this possible, and the ultimate goal is to dramatically improve the standard and satisfaction of living by providing quality services at affordable prices [6,7].

**Figure 1.**
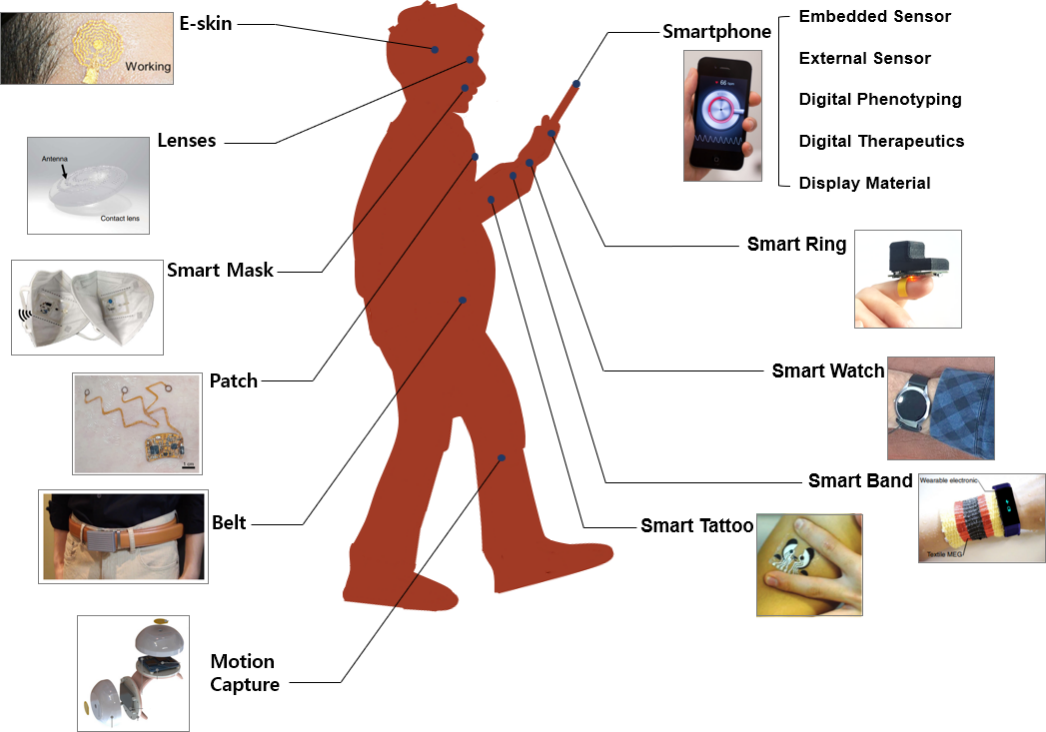
Application and placement schematic illustration of wearable devices by body part and smartphone for mHealth management. The images were reprinted from Shin et al [8,19], Kim et al [9,19], Escobedo et al [10,19], Hwang et al [11], Nakamura et al [12,19], Hua et al [13,19], Moon et al [14,19], Zhao et al [15,19], Kim et al [16,19], Liu et al [17,19], and Chan et al [18,19]. e-Skin: electronic skin; mHealth: mobile health.

Wearable electronics and smartphones are representative types of mobile systems optimized for personalized health care sensing. As shown in Figure 1 [8-19], wearable devices that cover the human body and smartphones, a necessity for modern people, enable comprehensive health management in real time.

However, the pace of the development and popularization of mHealth technology is progressing more slowly than expected. From the perspective of a paradigm shift from the smartphonization of wearable devices and the wearable deviceization of smartphones, this viewpoint aimed to propose ways to unleash the potentiality of mHealth in the postpandemic era. The smartphonization of wearable devices and the wearable deviceization of smartphones do not simply mean that current smartphones become wearable devices and that current wearable devices maintain the functions of current smartphones. The smartphonization of wearable devices is to completely replace the smartphone function with a wearable device, while upgrading health care performance by embedding the current smartphone’s computational power and sensor-integrated display, including large-area panels and user interaction, in the wearable system. In addition, the wearable deviceization of smartphones refers to a change in the form factor so that health care sensing can be performed by switching from the current rigid form to a form that can be attached to a curved skin surface. The new form factor, which features both wearable computer and smartphone functions, will improve detection performance through large-area sensing and increase the penetration rate.

This viewpoint investigated recent trends in health care sensing methods using wearable devices and smartphones, which are the central axis of mHealth. In the case of wearable devices, the form factor for each detailed location on the body and the corresponding detection target technology was described. In the case of smartphones, it covered the detection target and principles of health care according to the application of internal and external sensors, materials, and software. This viewpoint also analyzed the prospects of and current challenges in existing mHealth systems and considered new health care solutions using flexible displays for the convergence form factor of smartphones and wearables. The differentiating point was to consider the direction of mHealth from the perspective of a sensor-integrated and new form factor display. Ultimately, from a display perspective, solutions for the smartphonization of wearable devices and the wearable deviceization of smartphones will provide insight into the health care paradigm shift.

## Recent Progress in the Development of Wearable Electronics for Health Care

The primary classification of wearable electronics based on the attachment position can be divided into the face, upper body, limbs, and whole body. Wearable clothes all over the body can also be classified separately.

### Face

#### Head

**Figure 2.**
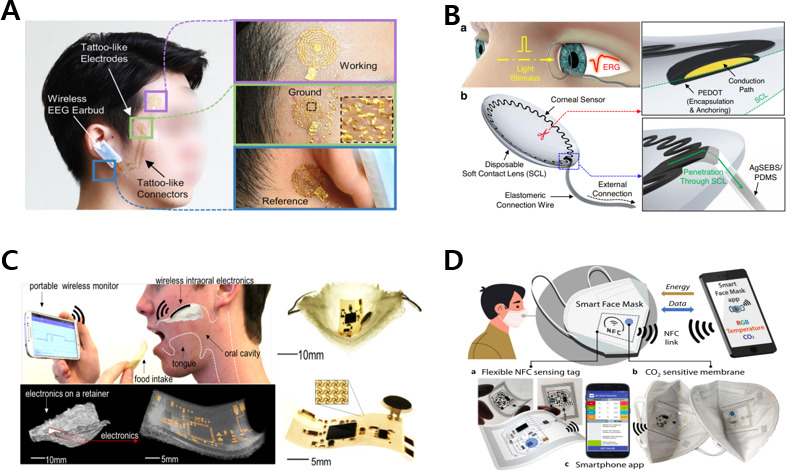
Wearable devices attached to the face for mHealth. (A) Wearable EEG analysis platform with tattoo electrodes for EEG measurement and earbuds for wireless interaction. The images were reprinted from Shin et al [8,19]. (B) Stretchable corneal lenses for ocular electrodiagnosis. The images were reprinted from Kim et al [19,22]. (C) Intraoral electronics for sodium intake analysis through wireless remote control. The images were reprinted from Lee et al [23]. (D) Sensing platform for gaseous CO2 real-time determination inside filtering face piece 2 (FFP2) facemasks. The images were reprinted from Escobedo et al [10,19]. EEG: electroencephalogram; mHealth: mobile health.

The face, which is closest to the brain, is significant from a sensory point of view because it is where the 5 senses are concentrated. Face-wearable devices with various form factors, such as bands, caps, headsets, lenses, glasses, tattoos, mouthguards, and masks, may be distributed at each part of the head, eyes, nose, mouth, and ears to sense critical biosignals. In the case of the head, a wearable system that can analyze brain waves and psychological states can be applied [8,20,21]. Figure 2A [8] shows a wireless wearable electroencephalogram (EEG) measurement device based on a tattoo. AI can enhance decision-making by deep learning classification of received EEG data. Namely, it advances the decision performance of AI by feedback through brain waves. Additionally, it would be possible to grasp the degree of brain activation and mental condition of the frontal lobe and temporal lobe through the measurement of biosignals, such as brain waves.

#### Eyes and Nose

System form factors worn on the eye may be divided mainly into lenses and glasses. In the case of lenses, eye health factors, such as glucose, intraocular pressure, and electroretinographic measurements, can be determined using noninvasive methods [9,22,24-26]. For example, a corneal sensor embedded in a disposable soft contact lens can be deployed for electroretinography based on electrochemical anchoring, as shown in Figure 2B [22]. These corneal lenses are functional sensors tailored for ophthalmic electroretinographic testing in human eyes via a user-friendly interface and a design that can be deployed noninvasively. Glasses for health care are prescribed by doctors as an auxiliary tool for surgery and can also analyze the electrolyte and metabolite content of sweat flowing from the head [27-29]. In addition, a wearable system placed on the nose in the form of a nose pad on the glasses can sense the pulse wave, respiratory rate, and electrooculographic measurements [30,31].

#### Mouth and Ears

Wearable electronics related to the mouth take the form of mouth guards, tooth sensors, and masts and can analyze saliva and nutrients and monitor air quality [10,23,32-35]. For example, a small stretchable circuit and sensor that can be inserted into the human oral cavity may be integrated into a breathable, flexible microporous membrane for a tissue-friendly design, as shown in Figure 2C [23]. Such a device may be used in research to study the prevention of hypertension by facilitating continuous quantification analysis of sodium intake. Figure 2D [10] shows a sensing platform for detecting gaseous CO_2_ inside a face mask via stable inorganic phosphors whose luminescence is controlled by a pH indicator. A mask combining a battery-free printed near-field communication (NFC) tag and a photochemical sensor for noninvasive CO_2_ measurement was used to achieve detection performance with a resolution of 103 ppm. Practicality in physical activity has been increased through the compensation of the temperature noise and characterized analytical specifications of measurement systems. Moreover, health care wearable systems attached to the ears use earbuds to perform heart rate and sleep monitoring functions [36,37].

### Upper Body

In addition to the face, wearable systems can be applied to the neck, chest, abdomen, internal organs, back, and waist to extract significant health values.

#### Neck

**Figure 3.**
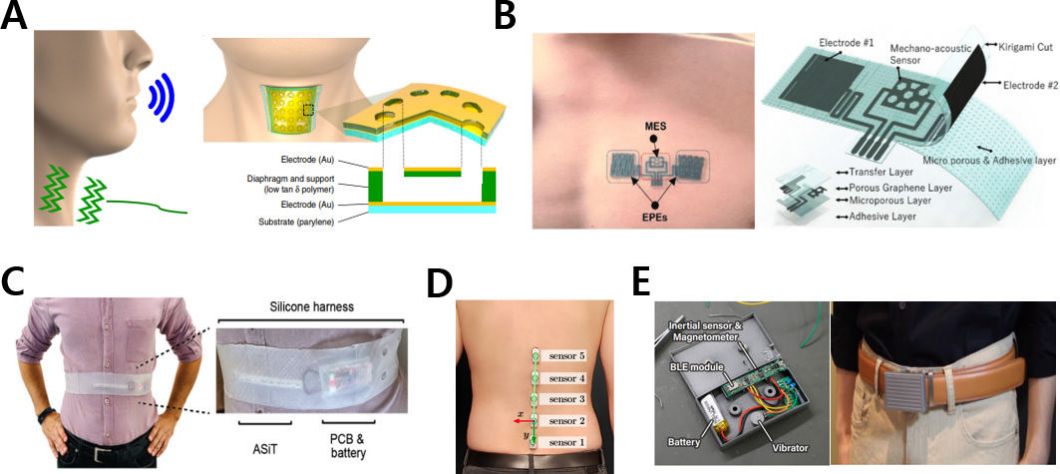
Wearable electronics mounted onto the upper body. (A) Vibration-responsive patch for sensing voice pressure. The images were reprinted from Lee et al [19,39]. (B) Epidermal cardiopulmonary patch based on laser fabrication. The images were reprinted from Rachim et al [41]. (C) Air-silicon composite transducer (ASiT) for breathing pattern monitoring. The images were reprinted from Cotur et al [19,42]. (D) Spine tracker sensor system. The image was reprinted from Stollenwerk et al [19,43]. (E) A belt for waistline measurement. The images were reprinted from Nakamura et al [12,19]. EPE: electrophysiological electrode; MES: mechano-acoustic sensor; PCB: printed circuit board.

In the case of the neck, wearable devices with a necklace and patch form factor can record an electrocardiogram (ECG) and voice pressure and monitor the diet through an electroglottogram (EGG) using a neckband [38-40]. For example, a neck-attached wearable device incorporating a cross-linked polymer film and hole-patterned diaphragm structure detects and quantifies voice with an excellent sensitivity of 5.5 V Pa^−1^ over the voice frequency range, as shown in Figure 3A [39]. This device can be used for voice health management and security authentication by eliminating vibration distortions on the curved skin surface through excellent skin compatibility via using ultrathin profiles of ≥5 µm.

#### Thorax

Thorax-related wearable electronics, such as patches, chest belts, and brassieres, enable ECG recording, temperature measurement, sleep monitoring, posture analysis, and galvanic skin response (GSR) assessment [11,41,44-48]. Figure 3B [41] shows a sensor designed for continuous monitoring of the cardiopulmonary biosignal via a CO_2_ laser–based manufacturing process. The epidermal patch consisting of a mechanoacoustic sensor and electrophysiological electrodes provides advanced functionality through a gas-permeable and biocompatible layer.

#### Abdomen

Abdomen-attached mHealth systems can sense glucose and breathing patterns through patches and straps [42,49]. For instance, an air-silicon composite transducer monitors respiratory activity by continuously measuring the force applied to the air channel embedded in the silicon-based elastomer, as shown in Figure 3C [42]. The system, which uses a pressure sensor and mixed-signal radio electronics, follows the principle of sensing the air pressure change inside the channel when breathing force is applied to the transducer surface. In particular, tactile sensing, including pressure sensing, is critical in health care. This is because tactile sensors attached to the skin detect physical stimuli, such as breathing patterns, heart rate, pulse, muscle activity, and body temperature, linked to biological signals. Skin, the most widely distributed organ among the five sense organs in the human body, is a tactile sensor with receptors that detect pressure, delicate movements, and temperature and is also an actuating organ that emits the same physical stimulation. Flexibility is a crucial element for the tactile sensor to be conformally attached to the skin to detect minute physical changes in detail and increase user convenience [50-52].

Furthermore, digestible pills check medication compliance. Management of medication adherence can prevent patients with severe mental illness from experiencing relapses and hospitalizations [53]. In addition, capsule endoscopy can monitor the colon health or bladder pressure state [54,55].

#### Back

A wearable system attached to the back can be used to analyze changes in the spine’s shape during training. A spine tracker device shown in Figure 3D consists of 5 sensors, with each sensor attached to the lumbar spine, and can correct posture by providing real-time feedback [43].

#### Waist

In addition, a waist belt can be useful for obesity management [12,56]. The belt automatically measures waist circumference with high accuracy, with an *F*_1_-score of 0.95, and monitors the daily lifestyle using a magnetometer, an accelerometer, and a gyroscope, as shown in Figure 3E [12].

### Limbs

In the case of the limbs, the main categories include the hands, arms, legs, and feet by attachment location.

#### Hands

**Figure 4.**
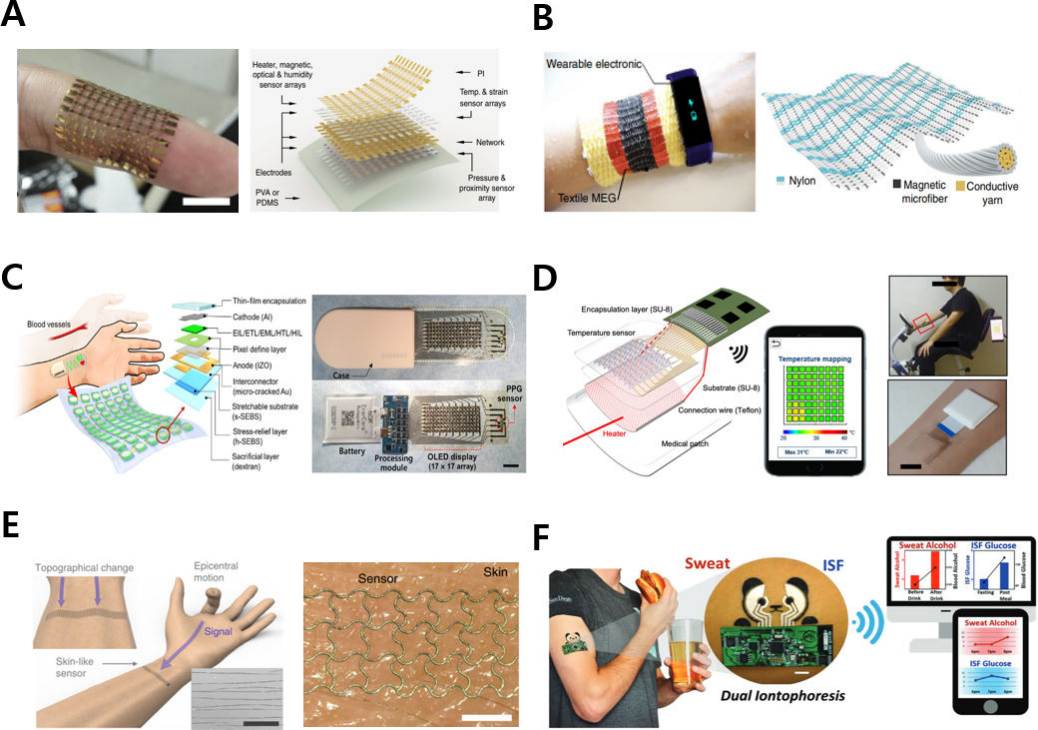
Wearable devices attached to the hands and arms. (A) Stretchable and conformable electronic skin for multifunctional sensing. The images were reprinted from Hua et al [13,19]. (B) Power generation textile for wearable health care. The images were reprinted from Zhao et al [15,19]. (C) Stand-alone patch for health monitoring based on a stretchable organic optoelectronic system. The images were reprinted from Lee et al [62]. (D) Thermal patch for self-care treatment through temperature distribution sensing and thermotherapy based on wireless graphene. The images were reprinted from Kang et al [63]. (E) Sensor conformably attached to skin decoding epicentral human motions. The images were reprinted from Kim et al [19,64]. (F) A single wearable biosensor platform that simultaneously monitors sweat and interstitial fluid (ISF). The images were reprinted from Kim et al [16,19]. MEG: magnetoelastic generator; OLED: organic light-emitting diode; PDMS: polydimethylsiloxane; PI: polyimide; PPG: photoplethysmogram; PVA: polyvinyl alcohol; Temp.: temperature.

The measurable health factors in a hand-related wearable device, such as a patch, ring, or glove, include rehabilitation evaluation analysis, ECG characteristics, oxygen saturation, dietary monitoring, pulse wave, and temperature [13,57-61]. For instance, a multisensory electronic skin integrated into a polyimide network simultaneously detects physical properties, such as temperature, strain, humidity, light, magnetic field, pressure, and proximity, in real time, as shown in Figure 4A [13]. It can also be used for rehabilitation evaluation using personalized intelligent prostheses.

#### Arms

mHealth systems of various form factors related to the arm can also be useful for health management. Among them, wristwatches, bands, and bracelet devices can detect health factors, such as the heart rate, oxygen saturation, number of steps, blood pressure, ECG characteristics, glucose, blood sugar, and sweat metabolites [14,15,65-75]. Figure 4B [15] shows a magnetoelastic generator that provides the power to drive a wearable biosensor system. This generator can help measure cardiovascular parameters underwater without encapsulation for telemedicine and has excellent water vapor transmission characteristics.

A patch sensor attached to the arm can measure the pH, sweat rate, lactate, heart rate, temperature, electromyogram (EMG) and ECG characteristics, blood pressure, and water content and can also be applied for wound treatment and rehabilitation evaluation [62-64,76-85]. For instance, a stand-alone organic skin patch for health care with an organic light-emitting display with sufficient pixels reports the heart rate via a stretchable photoplethysmogram (PPG) sensor, as shown in Figure 4C [62]. An ultrathin patch of 15 μm is configured on a soft elastomer substrate and can operate stably at 30% strain using a combination of a stress relief layer and deformable microcracks. Figure 4D [63] shows a wireless graphene patch that simultaneously provides thermal sensing and thermotherapy capabilities. This thermal patch consists of a graphene-based capacitive sensor, a graphene thermal pad, and a flexible wireless communication module to continuously monitor temperature changes with high resolution and sensitivity and perform thermal treatment through a graphene-based heater. Beyond the existing complex multisensor structure, skin patches alone may decode movements of 5-finger gestures by detecting microdeformation using the laser-induced crack structure, as shown in Figure 4E [64]. Based on the same principle, it can be attached to various body parts to track physical movements.

Furthermore, ECG, EMG, temperature, sweat, and interstitial fluid analyses can be performed following health care monitoring through arm tattoos [16,86]. For instance, a noninvasive epidermal biosensing system includes physically separated electrochemical biosensors for the extraction of interstitial fluid at the cathode and sweat stimulus extraction at the anode, as shown in Figure 4F [16]. Namely, this biomarker monitoring system is a single wearable epidermal platform that simultaneously samples and analyses different biofluids.

#### Legs and Feet

**Figure 5.**
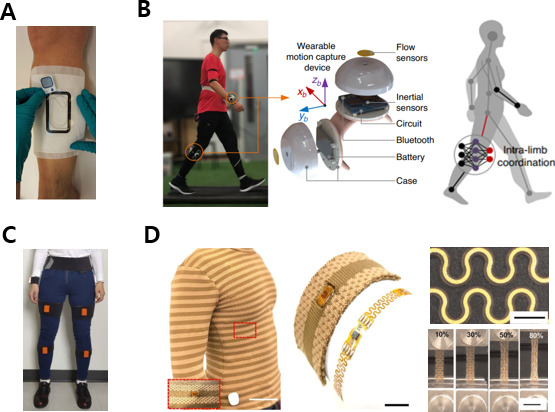
mHealth apps for the legs and the whole body. (A) Moisture sensor for exudative wounds. The image was reprinted from Henricson et al [19,89]. (B) A motion capture device capable of detecting limb movements with high accuracy. The images were reprinted from Liu et al [17,19]. (C) Wearable strap sensor for gait analysis. The image was reprinted from Luo et al [19,92]. (D) An electronic textile conformable suit for distributed sensing wirelessly. The images were reprinted from Wicaksono et al [19,98]. mHealth: mobile health.

Figure 5 describes a wearable health care device that may be applied to the legs, feet, or whole body. The mobile form factors applicable to the legs include patches, wearable robots, and straps, which perform moisture analysis at the wound area, gait analysis, ECG measurement, and rehabilitation evaluation [17,87-92]. For instance, appropriate dressing changes for exudative wounds are essential. Using a moisture sensor mounted on the bandage, as shown in Figure 5A [89], the change in the amount of dressing on the wound can be detected and the replacement time determined, increasing patient convenience. A motion capture device can accurately measure the movement of limbs during daily activities, strenuous exercise, and long-term exercise, as shown in Figure 5B [17]. Existing drift and instability problems are solved by integrating microtriaxis inertial and microtriaxis flow sensors. Additionally, it is possible to evaluate gait performance on irregular and uneven surfaces using a wearable sensor in the form of a strap with 6 inertial measurement units (IMUs) and an analysis algorithm, as shown in Figure 5C [92]. It is possible to implement edema measurement, gait analysis, and ulcer detection via plantar pressure analysis using wearable sensors attached to the shoes, socks, or soles of the feet [93-97].

### Whole Body

Furthermore, clothes worn on the whole body are also a type of wearable device. Figure 5D [98] shows a personalized and conformable suit of an electronics-based textile for multimodal health care sensing. The platform’s elasticity ensures intimate contact between the electronic device and the skin, and it can detect the skin temperature, heart rate, and respiration with high accuracy and precision. The suit with electronic textiles can measure the body temperature, respiratory rate, heart rate, oxygen saturation, and EMG and ECG characteristics and can also perform phototherapy [98-103]. As described before, form factor and detection targets by body part on wearable devices are summarized in Tables 1-4.

**Table 1.** Summary of form factor and detection targets on wearable devices for the face.

Body position and form factor	Target(s) of detection	Reference(s)
**Head**		
Tattoo	EEG^a^	[8]
Band, cap, headset	Mental stress through EEG	[20]
Band, cap, headset	EEG	[21]
**Eyes**		
Lenses	Glucose	[24,25]
Lenses	Intraocular pressure	[9,26]
Lenses	Electroretinogram	[22]
Glasses	Auxiliary surgical tool	[27,28]
Glasses	Sweat electrolytes, metabolites	[29]
**Nose**		
Nose pad	Pulse wave, respiration rate	[30]
Nose pad	Electrooculogram	[31]
**Mouth**		
Mouthguard	Saliva monitoring	[32-34]
Mouthguard	Nutrition analysis	[23]
Tooth sensor	Nutrition analysis	[35]
Mask	Air quality monitoring	[10]
**Ears**		
Earbuds	Heart rate	[36]
Earbuds	Sleep monitoring using EEG	[37]

a EEG: electroencephalogram.

**Table 2.** Summary of form factor and detection targets on wearable devices for the upper body.

Body position and form factor	Target(s) of detection	Reference(s)
**Neck**		
Necklace	ECG^a^	[38]
Patch	Voice pressure	[39]
Band	EGG^b^	[40]
**Thorax**		
Patch	ECG	[11,41,44]
Patch	ECG, temperature	[45]
Patch	Sleep monitoring	[46]
Chest belt	Trunk posture	[47]
Brassiere	Galvanic skin response	[48]
**Abdomen**		
Patch	Glucose	[49]
Strap	Respiratory patterns	[42,50-52]
**Internal organs**		
Ingestible pill/capsule	Medication compliance	[53]
Ingestible pill/capsule	Intravesical pressure and colon monitoring	[54,55]
**Back**		
Strap	Spine monitoring	[43]
**Waist**		
Belt	Obesity management	[12,56]

a ECG: electrocardiogram.

b EGG: electroglottogram.

**Table 3.** Summary of form factor and detection targets on wearable devices for the limbs.

Body position and form factor	Target(s) of detection	Reference(s)
**Hands**		
Patch	Rehabilitation	[13]
Ring	ECG^a^	[57]
Ring	SpO_2_^b^	[58]
Ring	Dietary management	[59]
Ring	Pulse wave, temperature	[60]
Glove	Rehabilitation	[61]
**Wrist**		
Watch/band/bracelet	Heart rate, step number	[65]
Watch/band/bracelet	SpO_2_	[66]
Watch/band/bracelet	SpO_2_, heart rate, energy expenditure	[67,68]
Watch/band/bracelet	Blood pressure	[14,69]
Watch/band/bracelet	Pulse management	[15]
Watch/band/bracelet	ECG	[70,71]
Watch/band/bracelet	Diagnosis of Parkinson disease	[72]
Watch/band/bracelet	Glucose	[73,74]
Watch/band/bracelet	Sweat metabolites (glucose, lactate)	[75]
Patch	Sweat rate, pH, lactate, glucose, chloride	[76]
Patch	Heart rate	[62]
Patch	Wound management	[77,78]
Patch	Temperature, thermotherapy	[63]
Patch	ECG, EMG^c^	[79-81]
Patch	EMG	[82,83]
Patch	Blood pressure, skin hydration, temperature	[84]
Patch	Biometrics	[85]
Patch	Rehabilitation	[64]
Tattoo	ECG, EMG, temperature	[86]
Tattoo	Sweat and Interstitial fluid analysis	[16]
**Legs**		
Patch	ECG	[87]
Patch	Moisture analysis at the wound area	[88,89]
Wearable robot	Rehabilitation	[17,90,91]
Strap	Gait analysis	[92]
**Feet**		
Patch	Edema	[93]
Shoes	Gait analysis	[94-96]
Socks	Foot pressure ulcer	[97]

a ECG: electrocardiogram.

b SpO_2_:oxygen saturation.

c EMG: electromyogram.

**Table 4.** Summary of form factor and detection targets on wearable devices for the whole body (clothes using electronic textiles).

Target(s) of detection	Reference(s)
Temperature, respiration, heart rate	[98]
SpO_2_^a^, heart rate, temperature	[99]
Phototherapy, temperature, heart rate	[100,101]
EMG^b^	[102]
ECG^c^	[103]

a SpO_2_:oxygen saturation.

b EMG: electromyogram.

c ECG: electrocardiogram.

## Recent Progress in the Development of Smartphone-Based Health Care Apps

In addition to wearable devices, health care delivery is also possible using smartphones through built-in sensors, smartphone-interlocked gadgets, display-related materials, and apps.

### CMOS Only

**Figure 6.**
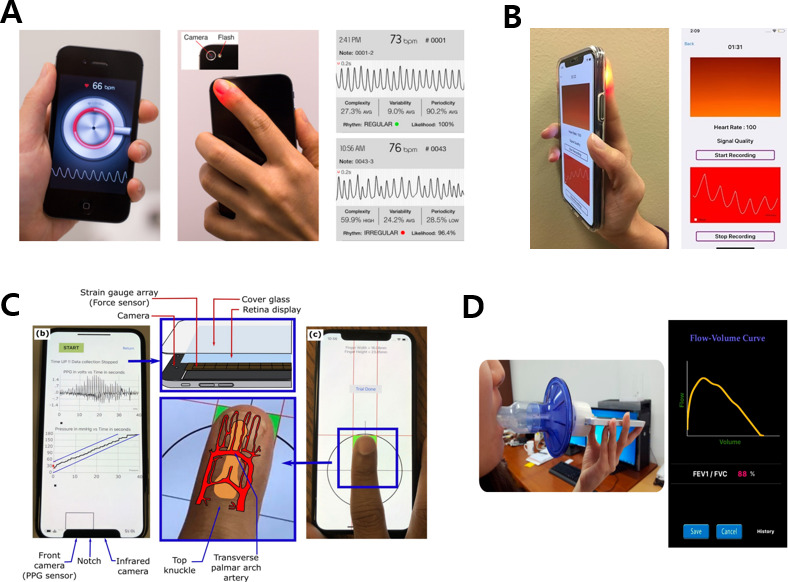
Health care apps using built-in smartphone sensors. (A) Smartphone built-in camera and app-based atrial fibrillation diagnosis. The images were reprinted from Chan et al [18,19]. (B) Heart rhythm analysis using CMOS image sensor. The images were reprinted from Tabei et al [19,104]. (C) Smartphone-based blood pressure measurement through the oscillometric finger-pressing method. The images were reprinted from Chandrasekhar et al [19,110]. (D) Set and acquisition graph of smartphone and 3D-printed mouthpiece adapter for spirometry. The images were reprinted from Thap et al [19,111]. CMOS: complementary metal-oxide-semiconductor; PPG: photoplethysmogram.

Smartphones have built-in 20-30 sensors; in particular, complementary metal-oxide-semiconductor (CMOS) image sensors may be used to monitor heart, eye, and skin-related diseases [18,104-109]. As shown in Figure 6A [18], the atrial fibrillation screening ability using PPG pulse analysis based on a smartphone camera and a commercialized app showed a similar performance level to that of patches used for single-lead ECG monitoring. It has been proven that prodromal stroke symptoms can be detected using only a smartphone in a primary care setting. In addition, the fingertip motion signal and color intensity signal, both heterogeneous signals, are acquired and analyzed using a camera to remove finger movement and optical noise, as shown in Figure 6B [104]. In this way, a clean heart rhythm signal with high accuracy can be extracted via smartphone monitoring, while minimizing noise artifacts.

### Hybrid Including CMOS

New functions, such as blood pressure measurement and temperature and dietary monitoring, can be established by combining pressure sensors, temperature sensors, and the phone microphone instead of CMOS alone [110,112-115]. For instance, as shown in Figure 6C [110], absolute blood pressure is measured via a blood flow oscillometric signal through finger pressure using a strain gauge on the front of the smartphone, in addition to CMOS. A light-emitting display may also be added to this, so it is possible to measure blood pressure ultimately with pure smartphone components.

### IMU/Microphone/Ultrasonic Sensor

In addition, sleep position monitoring and treatment can be performed by detecting body movements through an IMU of the smartphone, and the gait of patients with Parkinson disease can also be analyzed [116-118]. The smartphone’s built-in microphone sensor can also assess lung capacity and breathing sounds and monitor sleep [111,119-121]. Figure 6D [111] reports lung capacity and function parameter measurements following smartphone microphone–based, high-resolution time-frequency spectral analysis. A moisture-resistant ultrasonic sensor using polyvinylidene fluoride can be used for biometric authentication through fingerprinting [122].

### Touch Sensor/Digitizer

Moreover, general user interfaces, such as a touch sensor and digitizer, can also be used for health care purposes. For example, the heart rate can be checked by assessing capacitance changes according to the heartbeat with a capacitive touch sensor. The touch sensor is also helpful in diagnosing Parkinson disease through touch accuracy analysis [123,124]. In addition, a digitizer for writing can be applied to biometric authentication through handwritten signature recognition [125,126].

### Interlocked Gadgets

**Figure 7.**
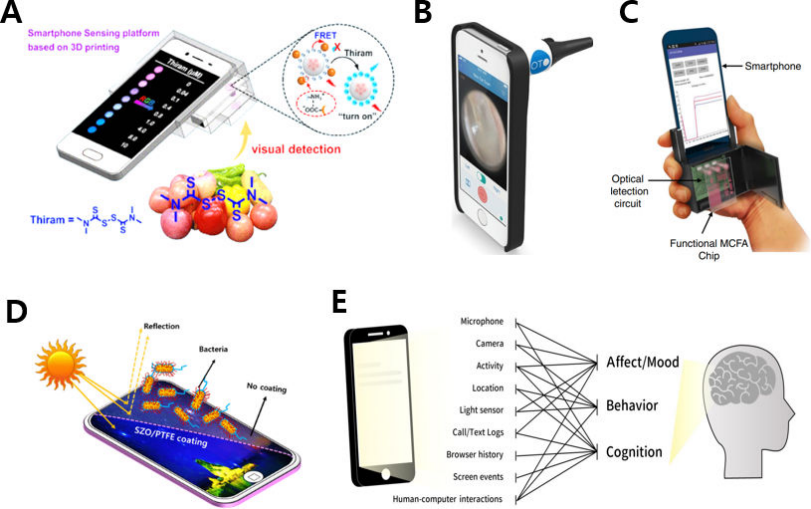
Health care apps using gadgets mounted on smartphones. (A) Smartphone platform for pesticide evaluation of food, integrated with an ultraviolet lamp and a dark cavity by 3D printing. The images were reprinted from Chu et al [128]. (B) Smartphone otoscope for diagnosis of acute otitis media. The images were reprinted from Mousseau et al [130]. (C) Smartphone-based immunodiagnosis using microfluidic assays. The images were reprinted from Ghosh et al [19,132]. (D) Antibacterial touchscreen for preventing contamination. The images were reprinted from Ippili et al [134]. (E) Digital biomarkers that reflect users’ moods, behaviors, and cognitions using text logs, browser history, human-computer interactions, and various sensors. The images were reprinted from Chen et al [19,135].

There is a case of combining various mHealth sensing techniques, such as pesticide analysis, otitis media diagnosis, malaria infection detection, and ECG measurement, by adding a separate gadget rather than using just the smartphone itself [127-133]. The platform shown in Figure 7A [128] performs a visual, quantitative analysis of pesticides using an optical system that combines a dark cavity and an ultraviolet lamp with a smartphone. In other words, integrating a smartphone and a gadget-based paper strip enables real-time and on-site food evaluation. Additionally, it was confirmed that the diagnosis of acute otitis media is possible with the same level of accuracy as that attained with existing otoscopes through the combination of a commercialized optical system and a camera in a smartphone, as shown in Figure 7B [130]. Figure 7C [132] shows a smartphone-based immunodiagnostic platform that performs a chemiluminescence-based enzyme-linked immunosorbent assay using a lyophilized chemiluminescence reagent. This hand-held point-of-care-testing analyzer can detect active malaria infections with a sensitivity of 8 ng/mL.

### Display Materials

Health care delivery can also be achieved through materials used in manufacturing smartphones, such as window coatings for antireflection and display processes. An ecofriendly antibacterial coating with Zn-doped silicon oxide thin films can prevent infectious diseases caused by microbial contamination of touch events, as shown in Figure 7D [134]. In addition, it is possible to reduce the deformation of retinal cells by decreasing the blue light of the display through the material development of organic light-emitting or color filters [136].

### Apps

Furthermore, health care sensing is possible through apps incorporating digital phenotypes and digital therapeutics [135,137-140]. A digital phenotype refers to a disease or health condition that is unintentionally reflected in patterns of use of digital devices. Mobile apps can collect human-smartphone interaction data to monitor smartphone usage and construct long-term patterns and trend changes. As shown in Figure 7E [135], analyzing a digital biomarker that reflects human effects, moods, behaviors, and cognition can predict psychiatric conditions, such as depression and smartphone addiction. In addition, digital therapeutics delivered through games, education, coaching, and counselling are based on cognitive behavioral therapy and can treat insomnia, alcohol addiction, drug addiction, panic disorder, and attention deficit hyperactivity disorder. Additionally, it effectively improves physical diseases, such as obesity and high blood glucose. Table 5 summarizes the sensing methods and targets using smartphones.

**Table 5.** Summary of sensing methods and targets using smartphones.

Type and sensing methods	Target(s) of detection	Reference(s)
**Built-in sensors**		
CMOS^a^	Atrial fibrillation	[18]
CMOS	Heart rate	[104,105]
CMOS	Diabetic retinopathy	[106]
CMOS	Skin cancer	[107-109]
CMOS + microphone	Heart rate, SpO_2_^b^, blood pressure	[112]
CMOS + microphone + speaker	Diet management	[113]
CMOS + strain gauge + display	Blood pressure	[110,114]
CMOS + temperature sensor	Temperature, heart rate	[115]
IMU^c^	Sleep monitoring	[116,117]
IMU	Gait analysis	[118]
Microphone	Spirometry	[111,119]
Microphone	Breathing sound analysis	[120]
Microphone	Sleep monitoring	[121]
Ultrasonic sensor	Biometric using fingerprint	[122]
Touch sensor	Heart rate	[123]
Touch sensor	Parkinson disease	[124]
Digitizer	Biometrics using signature	[125,126]
**Gadgets interlocked with smartphones**		
Optical platform	Pesticide evaluation in food	[127-129]
Smartphone CMOS + lens	Otoscopy	[130,131]
Microfluidic platform	Malaria infection	[132]
Patch electrode	ECG^d^	[133]
**Materials**		
Window coating	Antibacterial	[134]
Light emitting	Blocking of blue light	[136]
**Apps**		
Digital phenotyping	Addiction, attention deficit hyperactivity disorder	[135,137,138]
Digital therapeutics	Mental health	[139,140]

a CMOS: complementary metal-oxide-semiconductor.

b SpO_2_:oxygen saturation.

c IMU: inertial measurement unit.

d ECG: electrocardiogram.

## Prospects for mHealth

The industry of mHealth is expected to grow explosively in the future. In particular, the third generation of medicine and therapies that rely on novel solutions are emerging beyond the existing state of mHealth. Among them, bioelectronic medicine is a nonpharmacological treatment category that stimulates nerve functions with energy, such as electricity, light, and ultrasonic waves. This approach uses an electronic device that controls metabolic function to maintain homeostasis by regulating hormones [141]. To date, electroceuticals have been used for obesity, asthma, sleep apnea, brain tumors, epilepsy, and Parkinson disease and have shown substantial and significant therapeutic effects [142-144]. It is also one of the most innovative fields in medicine because it has significant advantages when considering the development time and cost of existing drugs.

Using digital therapeutics, also referred to as “software as a medical device,” it is possible to manage and treat not only physical diseases but also psychiatric conditions, such as posttraumatic stress disorder and schizophrenia [145,146]. It is of great significance in terms of patient convenience that personal and sensitive mental health conditions can be diagnosed in real life, not in hospitals, through digital phenotypic analysis, such as smartphone usage patterns and uploaded social networking service (SNS) content.

From the point of view of the wearable form factor, since much of health care sensing is possible on the wrist, the smartwatch is currently playing a pivotal role in health care. The finger (as well as the wrist) is a body part to focus on as it can be used to assess health factors, such as the heart rate, oxygen saturation, ECG characteristics, blood pressure, blood sugar, biometric authentication, body temperature, and dietary monitoring. Therefore, it is expected that in the future, the ring type of device for health care will pair with the smartwatch as the 2 main pillars.

## Challenges for mHealth

This viewpoint investigated comprehensive health care sensing technology using wearable electronics and smartphones. However, mHealth is less widely used than expected, unfortunately. Wearable devices are relatively more optimized for continuous and real-time health care sensing compared to smartphones [147,148]. However, the penetration rate compared to smartphones worldwide is sluggish [149-151]. A smartwatch, a representative wearable device, needs to be connected to a smartphone to operate, so users do not recognize the wearable device as an independent entity. Independent use is required to be fully positioned as a separate device. These devices lack effectiveness due to reduced user convenience because of their small screens, poor battery performance, low usage rate, clunky design, and high price. Wearable devices are recognized as a kind of subdevice rather than an essential and leading product because they do not have as much impact as smartphones. Therefore, in the case of wearable devices, innovative solutions are required to make them universal necessities for human beings, such as smartphones.

However, in the case of smartphones, the penetration rate is high worldwide, including low- and middle-income countries [152]. In the case of current smartphones, the fundamental value in terms of user experience as well as utility is high. However, it is not such a great solution from the perspective of health care. It is challenging to conduct biosignal sensing using a smartphone while being in close contact with human skin all day long, so it is challenging to implement continuous real-time big data–based predictive and preventive medical care using smartphones from the health care perspective. Smartphones desperately require a breakthrough that can allow them to monitor health in real time continuously, 24 hours a day, through a form more closely adherent to the skin, while maintaining the current phone function.

## Breakthroughs for mHealth

The display is a crucial component of a health care system. In other words, smartphones and wearable devices, as central axes of the mHealth system, are inseparable from their displays. In addition, displays and sensors in mobile devices are closely related. To improve the convenience of user interaction, the proportion of the active area of mobile displays is increasing. However, the increase in the active area has a limitation that reduces the sensing performance, including sensitivity. To overcome this, the upper part of the sensor covers the display by lowering the resolution of the display to prevent the deterioration of the sensing transmittance. A typical example is under-panel camera (UPC) technology that covers the camera with the display by reducing the display resolution on the top of the CMOS image sensor to increase light transmittance.

### Sensor-Integrated Display Solution

However, the ultimate and ideal method is a sensor-integrated display solution. A sensor-integrated display has many advantages from a health care sensing point of view. This is because (1) many mHealth sensors use an optical approach, (2) it is relatively easy to manufacture large-area sensors, and (3) the application of a new form factor display can lead to an increase in the body contact area.

First, the majority of mHealth sensing approaches are optical methods. Various health care parameters, such as the heart rate, oxygen saturation, blood pressure, blood sugar, body temperature, environmental monitoring, and ECH characteristics, can be measured optically. A display is an optical system that already has the means to transmit light. Therefore, a sensor-integrated display could be an optimized health care solution. To implement health care devices using optical systems, in addition to optical transmitters, receiver systems must also be equipped. For advanced performance, the light-emitting wavelength band needs to be expanded and supplemented, including infrared as well as visible light, through the development of materials for the light-emitting layer.

Second, since the sensing area and detection performance are proportional, health care ability can be improved through a sensor embedded in a wide display area. It enables health care sensing in a large area over the entire display area when the built-in optical system is applied, considering design rules. In addition, it is more advantageous for wearability because of a reduction in volume due to the implementation of microlevel thickness because of the sensor-integrated display. Additionally, compared to the number of photomasks needed to manufacture a conventional display, the number of additional photomasks required to implement a display health care system with built-in sensors is far less. It can contribute to popularization due to the low manufacturing price according to the integral type. Ultrathin, low-cost health care devices with relatively simple processes have significant benefits over conventional, bulky, and expensive wearable computers.

**Figure 8.**
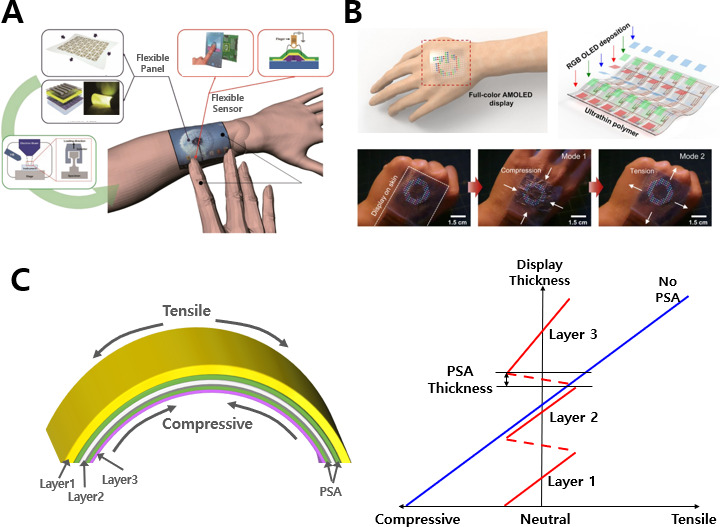
New form factor display and principle. (A) Wearable display with flexible and ultrathin active matrix backplane, touch screen panel, and fingerprint sensor components. The image was reprinted from Park et al [19,153]. (B) A flexible active matrix organic light-emitting diode (AMOLED) with large-area MoS2-based backplane for human skin display. The images were reprinted from Choi et al [154]. (C) A graph of compressive and tensile strength as the thickness increases in a single-layer structure (solid blue line) and laminated structure (dotted red line).

Finally, the new form factor device, such as a stretchable sensor-integrated display, increases the area of contact with the body and improves detection capability through health care sensing in close contact with the skin. Flexible panels with user convenience could be applied to the human skin, considering ergonomic factors [153-159]. The flexibility of not only the active matrix backplane and core of the panel but also the touch sensor, fingerprint sensor, and pressure sensor must be ensured, as shown in Figure 8A [153]. In a complete sensor-integrated display, the flexibility of the backplane allows the sensor part to gain flexibility naturally.

Furthermore, Figure 8B [154] shows a wearable full-color organic light-emitting diode (OLED) display using a 2D material–based backplane transistor suitable for complex skin shapes. The 18×18 thin-film transistor array was fabricated on ultrathin MoS_2_ film and then transferred to Al_2_O_3_ (30 nm)/polyethene terephthalate (6 μm), providing mechanical flexibility beyond conventional OLED technology.

### New Form Factor Display

The left picture of Figure 8C simulates a multilayered display, and when this display is bent, tensile strength is applied at the top and compressive strength is applied at the bottom. Assuming that it is formed with only a single layer of the same thickness rather than a laminated structure, extreme tensile and compressive forces occur on the upper and lower surfaces, resulting in cracks in the display, as shown by the solid blue line in the right graph. However, in the stacked structure, a pressure-sensitive adhesive (PSA) between the display layers continues to create new neutral planes, as shown by the dotted red line. In response, the magnitude of the tension and compression force at the top and bottom surfaces does not increase, even if the thickness of the display increases. In other words, using the PSA, it is possible to implement a flexible display without cracks.

No part of the human body is flat. When the health care system and the skin conformally adhere, sensing performance improves. Display technology based on PSA with the harmony of creep and recovery characteristics induces form changes in wearable devices and smartphones. A new form factor with flexibility based on PSA technology that creates a new neutral plane will facilitate a critical conversion of the mHealth system.

## Standard of the Medical Paradigm in the Postpandemic Era

A new form factor display for health care with flexibility and display convergence technology using an optical method attaches a large-area health care system to the human skin conformally and continuously detects health care factors in real time, thereby providing a framework for collecting big data. As a result, the existing smartphone becomes a wearable device attached to the body, and the existing wearable device is equipped with smartphone functions suitable for user convenience. Namely, convergence health care technology with the sensor-integrated and new form factor display is an indispensable element that enables the smartphonization of a wearable device and the wearable deviceization of the smartphone. Of course, health care systems with new form factors and sensor-integrated displays do not solve all mHealth problems. In other words, advances in big data AI software analysis and medical security should go hand in hand with the smartphonization of wearable devices and the wearable deviceization of smartphones. Furthermore, it will be necessary to supplement the medical system policy so that these benefits do not become the exclusive property of the upper class of the economy and so that people from lower social classes can also benefit. Advanced and popularized mHealth system technology could ensure universal health coverage so that everyone can use essential, high-quality medical services without discrimination. In other words, the authentic democratization of health care could become a reality, and a standard for a future health care paradigm in the post-pandemic era could arise.

## Conclusion

Personalized platforms, such as wearable devices and smartphones, can be applied to AI-based disease prediction, prevention, and treatment. This viewpoint researched the latest technology trends in mHealth regarding form factors and detection targets according to body attachment location and type. In particular, the sensor convergence technology of the new form factor display provides a framework to analyze health factors in real time by conformally adhering a large-area system to the skin. Innovation in form factors in sensor-integrated displays and convergence health care solutions enable the smartphonization of wearable devices and the wearable deviceization of smartphones. In addition, the strategy for the smartphonization of wearable devices and the wearable deviceization of smartphones can accelerate the development of mHealth, realizing the democratization of medical care so that anyone can use essential services of high quality. Furthermore, it is expected to create a new milestone for the medical paradigm shift in the postpandemic era.

## Abbreviations

AI: artificial intelligence
CMOS: complementary metal-oxide-semiconductor
ECG: electrocardiogram
EEG: electroencephalogram
EGG: electroglottogram
EMG: electromyogram
IMU: inertial measurement unit mHealth: mobile health
OLED: organic light-emitting diode
PPG: photoplethysmogram
PSA: pressure-sensitive adhesive
